# Increasing the fracture strength of MOD restorations with Ribbond fibers

**DOI:** 10.4317/jced.61608

**Published:** 2024-06-01

**Authors:** Francesca Zotti, Francesca Ferrari, Corrado Paganelli, Francesca Pilati, Giorgia Lanzaretti, Daniele Arlacchi, Nicoletta Zerman

**Affiliations:** 1 Researcher, PhD, Section of Dentistry and Maxillofacial Surgery, Department of Surgical Sciences, Pediatrics and Gynecology, University of Verona, P.le L.A. Scuro, 10, 37134 Verona, Italy; 2 DDS, Private Practice Verona, 37060; 3 Full professor, Department of Surgical Sciences, Paediatrics and Gynecology, University of Verona, 37134 Verona, Italy (IT); 4 Dental student, Section of Dentistry and Maxillofacial Surgery, Department of Surgical Sciences, Pediatrics and Gynecology, University of Verona, P.le L.A. Scuro, 10, 37134 Verona, Italy; 5 Associate Professor, Section of Dentistry and Maxillofacial Surgery, Department of Surgical Sciences, Pediatrics and Gynecology, University of Verona, P.le L.A. Scuro, 10, 37134 Verona, Italy

## Abstract

**Background:**

The aim of this study was to compare the fracture strength of two different Ribbond Fiber (Ribbond, Ribbond Inc., Seattle, WA, USA) restoration strategies in 5 mm deep standardized MOD cavities without interaxial dentin.

**Material and Methods:**

34 extracted human molars were randomly divided into two groups and restored as follows: Group 1 restoration with Ribbond Fiber placed at the cavity floor incorporated in Estelite Bulk-Fill Flow Universal composite (Tokuyama Dental Corporation Inc., Tokyo, Japan); Group 2 restoration with RF placed at 3 mm from the occlusal plane over a 2mm layer of Estelite Bulk-Fill Flow. The occlusal plane in both groups was restored with Ceram.x Spectra ST (Dentsply Sirona, Ballantyne Corporate Pl, Charlotte, NC, USA). The restored teeth were subjected to thermal cycling by immersing them for 30 seconds in hot water (55±2°C) followed by 30 seconds in cold water (5±2°C), for 2000 cycles.Their fracture strength was then evaluated using an Instron device. Data were analyzed with Two-sample T Test statistical test to compare fracture strength among groups. Finally, a descriptive analysis of the failure location was performed.

**Results:**

A statistically significant difference was found between groups 1 and 2 (*P*<0.001) in terms of fracture strength. Group 2 exhibited a higher percentage of recoverable fractures compared to group 1. Group 1 had a mean fracture load of 833N and a SD of 248 while group 2 had a mean fracture load of 1286N and SD of 447.

**Conclusions:**

RF placed at 3 mm depth from the occlusal plane, on a 2 mm layer Estelite Bulk-Fill Flow Universal composite (Tokuyama Dental Corporation Inc., Tokyo, Japan) contributes to improve fracture resistance in vital teeth without interaxial dentin and reduces the risk of non-recoverable fractures compared to when it is placed at a 5 mm depth.

** Key words:**Ribbond fiber, composite restoration, fracture resistance, Instron machine.

## Introduction

Fracture strength represents the maximum stress that a material can endure before breaking. To measure the strength of dental elements subjected to occlusal loads, particularly, compressive strength is evaluated. Fracture strength is a crucial property of restorative materials and can be linked to intraoral fractures of the restoration’s margins and surfaces. The study of the strength of dental elements restored with such materials is essential because each dental element is subjected to approximately 2000 loads per day, ranging between 30-80 kg, due to maximum intercuspidation contacts occurring during swallowing, alarm reaction, physical exertion, and parafunctions.

Several structural factors serve as indicators of the prognosis for restorations: the interaxial dentin, the pulp chamber roof, the marginal ridges, and the remaining cusp material (1). Interaxial dentin and marginal ridges are the most significant factors in terms of resistance, and it is essential to understand that these factors are interconnected. Consequently, the fracture strength of restorations is contingent upon the cavity’s morphology, with MOD (mesial-occlusal-distal) cavities being the most prone to fractures.

As observed in the analysis of structural factors of the tooth, the extent of the dental cavity, and especially its depth, are fundamental factors concerning the strength of the dental element. In an intact molar, the average fracture strength is approximately 3000 N, while in a molar with a Class I cavity restored with resin-based composite, the fracture strength ranges from around 1000 to 1900 N and reduces to 1600 N for elements with MOD restorations (2).

In particular, molars with MOD cavities 3 mm deep or less, restored with resin-based composites, have shown a similar strength to that of intact elements. Molars with MOD cavities restored with resin-based composites of a depth of at least 5 mm, on the other hand, show a significant difference in fracture strength compared to intact molars, regardless of wall thickness (3).

In addition to the well-established restorative materials, such as flowable resins and composites, which have undergone extensive investigation regarding their mechanical, aesthetic, and adhesive properties (4), fiber-reinforced composites have entered the market in recent years. These materials are composite resins strengthened through the incorporation of fibrous materials such as glass fibers, carbon fibers, Kevlar, Vectran fibers, and polyethylene fibers within the cavity, resulting in a substantial enhancement of the resins’ mechanical characteristics. Polyethylene fibers are the preferred choice in restorative dentistry due to their ability to augment the flexural strength, stress resistance, and modulus of elasticity of composite resins, all while delivering satisfactory aesthetic performance since they are scarcely perceptible when embedded in a resin matrix. Among these materials, Ribbond Fiber (RF) stands out as one of the most commonly utilized options. It comprises woven fibers made of high-molecular-weight polyethylene, boasting a high coefficient of elasticity (117 GPa), which translates to remarkable resistance against elongation and deformation, along with high tensile strength (3 GPa). These properties allow it to conform to the tooth cavity’s morphology effectively. Ribbond Fiber undergoes a cold plasma treatment that enhances its water absorption, reduces surface tension, and promotes improved chemical adhesion to composites (5). Ribbond fiber finds widespread application across various dental disciplines; however, there is a paucity of robust literature on its use in restoring vital teeth.

In a study previously published by Zotti F., the fracture strength of MOD composite resin restorations reinforced with Ribbond Fiber was investigated (6). The results of this study highlighted that Ribbond Fiber effectively demonstrates its reinforcing characteristics when the maximum cavity depth in the occlusal isthmus region does not exceed 3 mm; its peculiarity consists in enhancing the restoration’s fracture resistance and reducing the likelihood of fractures in the remaining dental tissue by absorbing forces more effectively at the composite resin restoration level. Conversely, in MOD cavities lacking interaxial dentin with a depth of 5 mm, the properties of Ribbond Fiber, when used as a cavity lining, appear to diminish, even though they still exceeded the results of 5 mm cavities restored without RF. Based on these findings, the intention to further explore the performance of Ribbond Fiber arises in the following experiment.

The first aim of this study was to compare the effectiveness of the reinforcement in terms of fracture strength of 5 mm-depths MOD restorations without interaxial dentin using RF (Ribbond, Ribbond Inc., Seattle, WA, USA). The latter was applied in two different modalities:

1. RF applied at the base of the cavity

2. RF applied on 2 mm of Bulk-Fill Flowable composite, specifically at a depth of 3 mm relative to the occlusal plane.

Furthermore, a descriptive assessment of the types of fractures occurring in the two groups was carried out.

## Material and Methods

The determination of the sample size was performed using the statistical software G-Power v. 3.1 (University of Düsseldorf; Düsseldorf, Germany). A significance analysis revealed that a sample size of 33 met the constraints of α = 0.2 and power = 0.95. To ensure equity between the two groups, 34 intact upper and lower molar teeth were collected. Teeth with both caries-free and Class I, II, and MOD carious lesions were included. Teeth with radicular carious lesions (lesions extending beyond the enamel-cement junction) and lesions leading to pulp exposure were excluded. All teeth were cleaned and disinfected with a 0.20 chlorhexidine solution for 60 seconds, washed, and kept in physiological solution for a period not exceeding 3 months (6).

The teeth were randomly divided into two groups and prepared as follows: 34 molars were prepared with MOD cavities measuring 5 mm in depth and a width that allowed for a maximum thickness of vestibular and lingual walls of at least 2.5 mm, assessed with a measuring caliper (Iwanson, Proclinic Italia, Italy). For cavity creation, a cylindrical diamond bur with a 1 mm diameter and a 5 mm working surface was used (836KR Burs, Komet, Germany). Subsequently, beveling of the margins and removal of unsupported enamel prisms were carried out using a 1 mm round-ended diamond bur (801.314 Bur, Komet, Germany). The teeth of group 1 (n= 17, G1) were restored with composite resin using Ribbond Fiber (Ribbond Inc., Seattle, WA, USA) placed at the base of the cavity. The teeth of group 2 (n= 17, G2) were restored with composite resin using Ribbond Fiber (Ribbond Inc., Seattle, WA, USA) placed over 2 mm of Estelite Bulk-Fill Flow Universal composite (Tokuyama Dental Corporation Inc., Tokyo, Japan) .

Conventional adhesive procedures, which were the same for both groups, were performed as follows: selective acid etching with Tokuyama Etching Gel HV (Tokuyama Dental Corporation Inc., Tokyo, Japan): 30 seconds on enamel and 15 seconds on dentin; rinse for 30 seconds with water; adhesive procedure using Tokuyama EE Bond (Tokuyama Dental Corporation Inc., Tokyo, Japan); polymerization for 30 seconds.

Preparation, by cutting with a scalpel, of a strip of Ribbond polyethylene fiber measuring 3 mm in width and equal in length to the mesio-distal dimension of the cavity floor. Saturation of RF with adhesive resin (Tokuyama EE Bond, Tokuyama Dental Corporation Inc., Tokyo, Japan) and removal of excess using a piece of absorbent paper without polymerization.

Restorations were performed as follows (6):

-Group 1.

oApplication of a thin layer (maximum 1 mm) of Estelite Bulk-Fill Flow Universal composite (Tokuyama Dental Corporation Inc., Tokyo, Japan) at the base of the cavity where the Ribbond strip is incorporated, extending mesio-distally;

oPolymerization for 20 seconds;

oRestoration by a micro incremental technique of Ceram.x Spectra ST (Dentsply Sirona, Ballantyne Corporate Pl, Charlotte, NC, USA);

oPolymerization for 30 seconds;

oFinishing and polishing were routinely performed.

-Group 2.

oApplication of a 2 mm layer of Estelite Bulk-Fill Flow Universal composite (Tokuyama Dental Corporation Inc., Tokyo, Japan) at the base of the cavity, placing the Ribbond strip (Ribbond, Ribbond Inc., Seattle, WA, USA) in the mesio-distal direction while ensuring there are no gaps between Ribbond and composite;

oPolymerization for 20 seconds;

oRestoration by a micro incremental technique of Ceram.x Spectra ST (Dentsply Sirona, Ballantyne Corporate Pl, Charlotte, NC, USA);

oPolymerization for 30 seconds;

oFinishing and polishing were routinely performed.

Consequently, all the restored specimens underwent thermal cycling. The authors chose to increase the total number of cycles to which the samples were subjected (compared to the ISO 11450 protocol of 2015), while maintaining the temperature ranges specified by the ISO TR 11450 protocol (5±2°C and 55±2°C). A total of 2000 cycles were conducted, a value that has been previously used in recent literature to test composite resins and simulate an aging process equivalent to approximately 3 months (7)). The samples were subjected to an immersion protocol of 30 seconds in hot water (55±2°C) followed by 30 seconds in cold water (5±2°C), totalling 2000 cycles (7). The time was measured using a digital chronometer (iPhone clock application, Apple Inc., USA). The cycles were completed in 16 hours and 40 minutes.

**Figure 1:**
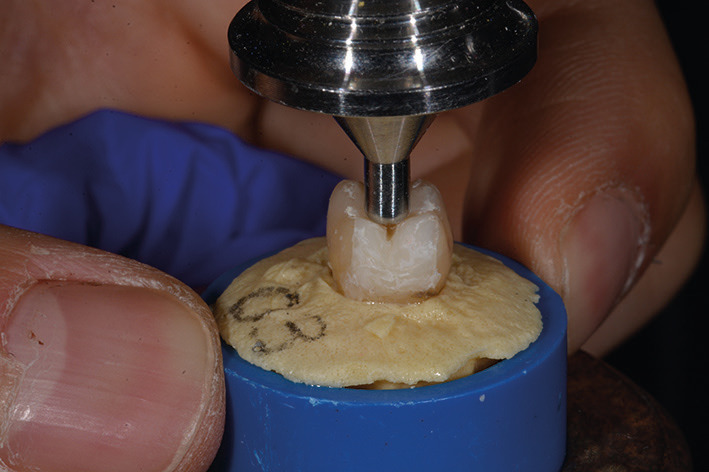
Insert with a 3 mm spherical tip.

The specimens were positioned within PVC containers measuring 20 mm in diameter and 20 mm in height,; a self-curing acrylic resin (SR Ivolen Kit - Ivoclar Vivadent, Liechtenstein) was poured up to the cement-enamel junction (CEJ) to secure them in place. These containers were then situated on the platform of the Instron Machine (Instron 5848, Norwood, Massachusetts USA), equipped with a 2000N load cell, for testing fracture strength under vertical axial loads. To ensure optimal contact between the fossa-cusps and the Instron tip, a specialized insert with a 3 mm spherical tip was designed and custom-manufactured (Fig. 1) (5).

As performed in a previous study (6), the machine was set to move within a predetermined vertical range of 2.5 mm in 90 seconds, with vertical loading of the occlusal surface at a rate of 0.028 mm/s. The following data were recorded:

-Load (Newton): Impressed force required to fracture the teeth during the test;

-Vertical area (mm): area in which the fracture occurs.

**Figure 2:**
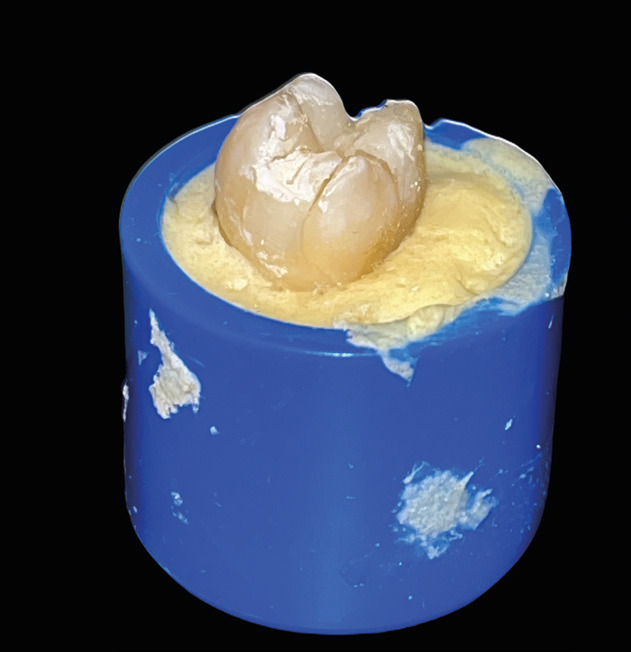
Example of fracture of the composite resin.

**Figure 3:**
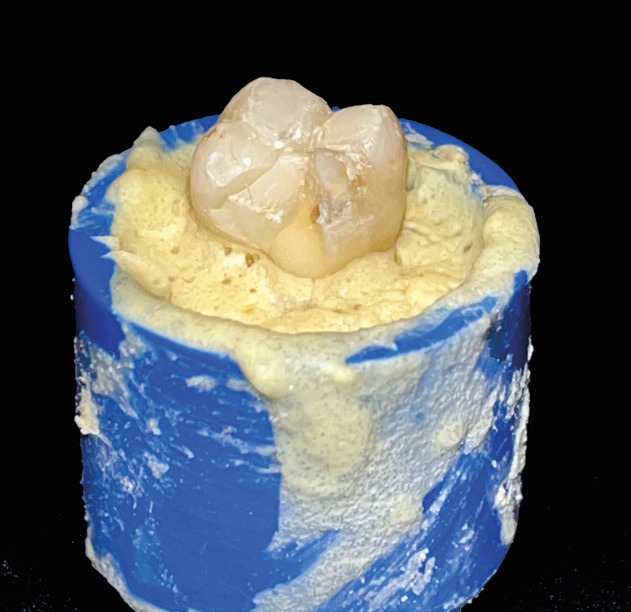
Example of fracture of one or more cusps.

**Figure 4:**
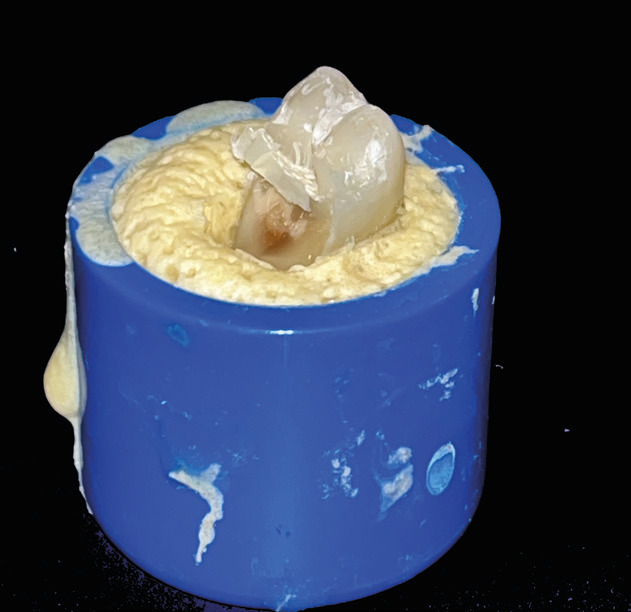
Example of fracture extending to the CEJ or deeper.

-Pattern of fracture:

1) Fracture of the composite resin (Fig. 2)

2) Fracture of one or more cusps (Fig. 3)

3) Fracture extending to the CEJ or deeper (Fig. 4)

Fractures of types 1 and 2 were considered recoverable, whereas type 3 fractures were classified as non recoverable.

-Statistical Analysis

The data were collected using the Instron software (Instron 5848, Norwood, Massachusetts USA) in a Table where each time value is associated with a specific applied load and a particular insert displacement. From this data, the Newton values of the fracture points for each tested element were extracted for analysis. The analysis considered the first Newton value at which the dental element exhibited structural failure as a reference point.

The data were tested for normality using the Shapiro-Wilk test.

The Two-sample T Test statistical test was employed to examine differences between the fracture points of elements in Group 1 (Ribbond at the base of the cavity) and Group 2 (Ribbond 3 mm from the occlusal plane).

For the analysis of the collected data, STATA18 software (StataCorp, 1985, California, USA) was utilized. Tests were considered significant for *p* ≤ 0.05.

The types of fractures (1,2,3) were analysed descriptively.

## Results

The Two-sample T Test revealed a statistically significant difference between groups 1 and 2 (*P* = 0.0009) regarding the load force required to achieve fracture. Additionally, upon descriptive analysis of the means of the two groups, it was observed that group 2 exhibited an average fracture strength of 1286 N compared to 833 N in group 1. Therefore, this leads to the conclusion that restorations in which Ribbond Fiber (Ribbond Inc., Seattle, WA, USA) was positioned 3 mm from the occlusal plane exhibit significantly higher fracture resistance compared to restorations where it was placed 5 mm deep.

In group 1, only 11.8% of the samples were found to have a recoverable type of fracture that could potentially be addressed with a second conservative intervention. In contrast, in group 2, this percentage increased to 41.15%. Specifically, fractures involving only the composite (Type 1) accounted for 5.9% of the samples in group 1 and 23.5% in group 2. Fractures of one or more residual cusps without exceeding the CEJ (Type 2) were observed in 5.9% of the elements in group 1 and 17.65% in group 2. Regarding fractures classified as non-recoverable (Type 3), the highest percentage was found in group 1, where they constituted 88.20% of cases, as opposed to 58.85% in group 2.

Results are shown in  Table 1 and  Table 2.

## Discussion

This study was conceived to assess whether RF (Ribbond Fiber) is capable of exerting its reinforcing properties in 5 mm MOD cavities devoid of interaxial dentin when placed at a depth of 3 mm. This idea stemmed from the conclusions of a study conducted by Zotti F. in 2023 (6). In this study, it was observed that RF effectively demonstrated its characteristics when the maximum depth of the cavity did not exceed 3 mm and interaxial dentin was retained. Conversely, the reinforcement with RF was found to lack significant efficacy in 5 mm cavities lacking interaxial dentin. This raised doubts as to whether positioning RF 2 mm from the cavity floor in a deep cavity without interaxial dentin could yield results closer to those achievable when interaxial dentin is present.

Both upper and lower molars were utilized in this study. These teeth are known to exhibit the highest degree of cuspal flexure under load in relation to the degree of residual dental tissue (5). The cavities were prepared to achieve a 5 mm mesio-occluso-distal depth, with interaxial dentin removed and a width sufficient to allow for the insertion of RF. Care was taken to preserve the roof of the pulp chamber and at least 2.5 mm of residual cuspal thickness on vestibular and lingual walls. In this study, we chose to restore dental elements while maintaining the integrity of the pulp chamber, a choice not always commonly practiced in the literature. However, we believed it would be interesting to study the fracture resistance of vital elements, albeit extensively restored, compared to endodontically treated elements, which often undergo indirect restorations or means aimed at reinforcing their integrity (such as endodontic posts or indirect restorations) (8). The ability to reinforce a dental element that has lost a significant amount of healthy structure, even in a phase of direct restoration, and to potentially ensure its integrity or restorability for a longer period, is a foundational goal of conservative dentistry. A constant thickness of residual cusps, despite efforts to maintain consistent measurements in all preparations, was sometimes not achievable due to anatomical variations of the samples. However, as noted by Forster (3), a minor difference in cuspal thickness does not significantly influence fracture resistance in directly restored teeth with MOD cavities.

In order to simulate the changes occurring in the oral cavity (9-12), the samples underwent thermocycling. This is a tooth aging system that, through repeated cycles of exposure to cold water (5±2°C) and hot water (55±2°C), induces stress at the composite-tooth interface and affects the marginal integrity of the restoration (13). Depending on the number of cycles performed, different aging times can be achieved. A thermocycling process of 500 cycles, as utilized in the study by Zotti F. (6), simulates dental exposure in the oral cavity for approximately 20 days (7). This cycle count is widely employed in dental research, although, according to some authors (6-9), it may be insufficient to represent an adequate aging period for the sample. Therefore, in this study, restored teeth underwent a 2000-cycle thermocycling process, which corresponds to an aging period of approximately 3 months (90 days) (7). Studies by Carreira and colleagues (14) indicate that it is particularly necessary to exceed 1500 cycles since physical and mechanical properties of composites do not significantly alter at such values. This choice represents an enhancement in the design of this study.

The specimens were subjected to a continuous load using an Instron machine. To achieve this, a specific insert designed by authors (6) was utilized. This insert is characterized by its spherical shape and a diameter of 3 mm, features that allow for maximum contact with the cusps and fossae of the teeth. The choice of using this insert was based on the intention to recreate a situation as similar as possible to the natural contact that occurs between the fossa and the opposing cusp. The selection of the insert used for fracture resistance testing with the Instron machine is also an important factor to consider when comparing test results with those of similar studies. As observed by Watts and colleagues (15), the difference in resistance is directly related to the diameter of the insert used: the larger the diameter, the higher the fracture resistance value. Teeth are continuously subjected to stress during normal oral functions, and prolonged cyclic occlusal loads can lead to cuspale displacements and incomplete restoration even after the removal of the applied load (14). Therefore, the fact that the samples in this study were subjected to a continuous load without considering cyclic loading may represent a limitation.

The values in terms of fracture resistance in N obtained from the experimentation are in line with the data observed in the literature for molars subjected to axial load (16). Values among different studies in the literature can vary significantly due to various factors: materials used, restoration technique, clinician experience, whether thermocycling was performed or not, sample preservation and integrity (17-19). Additionally, as mentioned earlier, much also depends on the type and size of the insert for the Instron Machine used (20).

The high standard deviation (SD) of fracture load present in this research, as also observed by Watts and colleagues in their study (21), is typical of mechanical tests on anatomically irregular samples, such as the occlusal surface of the tested molars.

This study was developed by creating two groups. In the first group, RF was applied at the base of the cavity, while in the second group, it was placed on a 2 mm layer of Estelite Bulk-Fill Flow Universal, which is 3 mm deep from the occlusal plane.

The results obtained in this experimentation are consistent with the findings of Zotti F. (6). However, it can be observed that in the groups of the two studies where RF is placed at a depth of 3 mm from the occlusal plane, there are differences both in the average fracture loads obtained and in the percentage of recoverable fractures that occurred. In the previous study (6), where RF was placed on interaxial dentin, the average fracture load was 1725.2 N, and the percentage of recoverable fractures was 80%. This difference may be attributed to the complete absence of interaxial dentin in the second group of this study. Interaxial dentin is indeed the most important structural factor, and its loss results in a significant weakening of the tooth (20-22). Therefore, despite the vertical forces acting on the tooth being partially absorbed and redistributed by fibers, some of these loads generate lateral forces against the cavity walls, which then create a tensile force directly on the roof of the pulp chamber. This can be responsible for the initial cracking of the remaining walls, and if the fracture starts from the dentin portion, after a certain degree of plastic and elastic deformation, it can extend even beyond the CEJ (23). However, it is important to consider the small sample size of the previous published study (6), as well as the higher number of thermocycling cycles undergone by the samples in this experimentation, which led to greater alteration of the dental tissue in these samples. Therefore, the data collected from this study suggest that RF may be considered a suiTable reinforcing material in clinical practice, even in 5 mm deep MOD cavities without interaxial dentin, when placed 3 mm from the occlusal plane. However, it would be interesting to study the role of the 2 mm thickness of Estelite Bulk-Fill Flow Universal in improving the fracture resistance of restored elements and reducing type 3 fractures in Group 2. Low-viscosity resin composites (Flowable) used as cavity liners can help reduce microleakage, polymerization shrinkage, and improve the distribution and absorption of forces exerted on the restored tooth (16). Furthermore, increasing the thickness of the lining within a certain range, appears to be correlated with a lower likelihood of mechanical stress formation (16). In particular, bulk-fill flow composites, when used in thicknesses up to 3 mm, exhibit fracture resistance comparable to that of restorations performed with traditional composites and unprepared teeth, while also reducing the incidence of enamel fractures and fractures below the CEJ (24-27). Therefore, in the future, further studies may be needed to investigate the use of Ribbond Fiber in 5 mm deep MOD cavities without interaxial dentin, comparing restored elements using only 2 mm of Bulk-Fill Flowable composite with elements restored with 2 mm of Bulk-Fill Flowable composite plus Ribbond Fiber.

## Conclusions

The data from this study demonstrate that RF (Ribbond Fiber) contributes to an improved prognosis for teeth that have lost significant amounts of dental tissue. The findings suggest that when placed 3 mm deep from the occlusal plane, on a 2 mm layer of Estelite Bulk-Fill Flow Universal composite, RF is capable of reinforcing dental structure even in 5 mm deep cavities without the presence of interaxial dentin, thereby enhancing the fracture resistance of the restoration. Furthermore, placing RF at a 3 mm depth reduces the risk of non-repairable fractures compared to when it is placed at a 5 mm depth.

## Figures and Tables

**Table 1:** Presents the mean fracture loads (N) of the two groups along with their respective standard deviations.

Groups	Mean fracture loads (N)	Standard deviations
Group 1	833.058	248.298
Group 2	1286.900	447.800

**Table 2:** Displays the distribution of fracture types that occurred in individual samples.

Groups	Type 1	Type 2	Type 3
Group 1 (n=17)	1 (5.90%)	1 (5.90%)	15 (88.29%)
Group 2 (n=17)	4 (23.50%)	3 (17.65%)	10 (58.85%)

## Data Availability

The datasets used and/or analyzed during the current study are available from the corresponding author.
